# High-Temperature Oxidation Behavior of Ti-Doped SiOC Ceramics

**DOI:** 10.3390/ma19020355

**Published:** 2026-01-16

**Authors:** Xiumei Wu, Xiaojuan Gong, Yunping Li, Xiangming Chen, Shu Yu

**Affiliations:** 1 National Key Laboratory of Science and Technology on High-Strength Structural Materials, Central South University, Changsha 410083, China; 2 Faculty of Mechanical & Materials Engineering, Huaiyin Institute of Technology, Huai’an 223003, China; xiaojuangong@hyit.edu.cn; 3 State Key Lab for Powder Metallurgy, Central South University, Changsha 410083, China; 4 Research and Development Center, Zhuzhou Cemented Carbide Cutting Tools Co., Ltd., Zhuzhou 412007, China

**Keywords:** SiTiOC ceramic, oxidation resistance, composite oxide layer, outward diffusion of Ti

## Abstract

Silicon oxycarbide (SiOC) ceramics are prone to failure prematurely in high-temperature applications for thermal stress-induced cracks. Doping Ti into SiOC can improve the oxidation resistance by forming a SiO_2_-TiO_2_ composite oxide layer. In this study, the oxidation behavior of Ti-doped SiOC ceramics in air at 1500 °C for 32 h was examined comprehensively. SiTiOC ceramics with a titanium-to-silicon molar ratio of 0.05 demonstrated the best oxidation resistance. The oxide layer was enhanced by the distribution of TiO_2_ and TiSiO_4_ at the grain boundaries of SiO_2_, which reduced the interfacial energy and inhibited crack propagation. Furthermore, the oxide layer composed primarily of SiO_2_ and minor TiO_2_ exhibited low oxygen diffusion coefficients and strong self-healing capability. However, increasing the titanium-to-silicon molar ratio to 0.2 generated many pores and cracks in the oxide layer, and the outward diffusion of Ti and active oxidation of TiC were been exacerbated during oxidation.

## 1. Introduction

Silicon oxycarbide (SiOC) ceramics have attracted considerable attention as high-temperature structural materials due to their excellent thermal stability, creep resistance, and crystallization resistance [1,2,3]. These advantageous properties make them suitable for various applications in aerospace (e.g., thermal barrier coatings [4,5]), energy storage (e.g., lithium-ion anodes [1]), and electronic sensors [6]. However, the high-temperature applications of SiOC ceramics are limited by the phase separation of the SiOC network above 1200 °C, the carbothermal reduction of SiO_2_ above 1400 °C [7,8,9], and high-temperature oxidation decomposition.

To overcome these disadvantages, extensive research has been conducted on the incorporation of metallic dopants, such as Al [10,11], Y [12], Zr [13,14,15], Hf [16,17,18], and Ti [7,19], into SiOC systems. Doping Al, Y, Zr, and Hf can form refractory oxides, which can effectively mitigate the phase separation of SiOC and the carbothermal reduction of SiO_2_, thereby enhancing thermal stability. Furthermore, the refractory oxides and their resultant silicates can enhance the oxidation resistance of ceramics by forming a composite oxide layer. For example, Zhang et al. reported that Zr-doped SiOC exhibited a substantial reduction in mass loss from ~6.5% to ~1.5% when heated from 25 °C to 1200 °C under a N_2_ atmosphere [15]. Sun et al. found that the Hf-doped SiOC demonstrated exceptional oxidation resistance during 15 h of oxidation in air at 1500 °C, including a formed continuous cristobalite-based oxide scale reinforced by HfO_2_ and HfSiO_4_ particles [18].

However, a new challenge has emerged as the refractory oxides in SiOC may react with free carbon, compromising structural integrity [19]. Fortunately, incorporating Ti into SiOC can in situ form a TiC phase, which inhibits the carbothermal reduction of SiO_2_ [19] and is expected to further improve thermal stability and oxidation resistance. Yang et al. reported that SiTiOC ceramics pyrolyzed at 1200 and 1300 °C exhibited superior stability in air from room temperature to 1000 °C, with a mass loss of 0.2% [7]. Nonetheless, the long-term oxidation behavior of Ti-doped SiOC at high temperatures (>1000 °C) and the quantitative relationship between the Ti content and the evolution of the oxide layer have yet to be systematically established.

In this work, SiTiOC ceramic bulks were prepared using spark plasma sintering (SPS). The influence of the Ti doping content on the oxidation behavior of SiTiOC ceramics in air at 1500 °C is systematically discussed, and the antioxidation mechanisms were also thoroughly analyzed. This research provides significant insights for development of Ti-doped SiOC ceramics for high-temperature applications.

## 2. Experimental Methods

### 2.1. Preparation of SiTiOC Ceramic Bulks

The synthesis of the SiTiOC ceramic powders involved a sol–gel process followed by pyrolysis, as reported in our prior research [20]. The precursor materials—dimethyldimethoxysilane (Aladdin, Shanghai, China) and methyltrimethoxysilane (Aladdin, Shanghai, China) with a molar ratio of 1:2—were first dissolved in anhydrous ethanol (Tianjin Hengxing, Tianjin, China). To initiate hydrolysis, deionized water acidified with hydrochloric acid (Hengyang Kaixing, Hengyang, China) to a pH of 4 was added under continuous stirring, forming a homogeneous SiOC prepolymer sol. Tetrabutyl titanate (Aladdin, Shanghai, China) was then introduced as a titanium source, with its amount adjusted to achieve final Ti/Si molar ratios of 0, 0.05, 0.1, and 0.2, yielding the SiTiOC precursor sol. This sol was subsequently gelled and dried at 80 °C to obtain polysiloxane xerogels. Finally, these xerogels were converted into ceramic powders via pyrolysis at 1400 °C for 2 h under a flowing argon atmosphere in a corundum tube furnace.

The synthesized ceramic powders (particle size < 20 μm) were then densified via spark plasma sintering (SPS, FCT HP D 25, FCT System GmbH, Frankenblick, Germany). For each batch, approximately 3.5 g of powder was loaded into a graphite die. Sintering was conducted at 1400 °C under a uniaxial pressure of 40 MPa, with a holding time of 10 min. This process produced fully dense ceramic bulks with a diameter of 20 mm and a thickness ranging from 2.8 to 3.2 mm. The resulting bulk samples were labeled 0Ti, 0.05Ti, 0.1Ti, and 0.2Ti, corresponding to their nominal Ti/Si molar ratios.

### 2.2. Characterization of SiTiOC Ceramics

The bulk density of the sintered SiTiOC ceramic was determined via the Archimede (water immersion) method. The phase identification of ceramics, both before and after oxidation, was conducted using an X-ray diffractometer (XRD, Advance D8, Bruker, Fällanden, Switzerland) with Cu-K_α_ radiation (λ = 0.15418 nm). XRD patterns were recorded over a 2θ range from 10° or 15° to 80° at a scan speed of 6°/min, with the instrument operating at 40 kV and 30 mA. Phase identification of the XRD patterns was conducted using Jade software (version 6.2). The chemical structure of ceramic samples was investigated with a Raman spectrometer (Raman, LabRam Aramis, HORIBA, Longjumeau, France) with a 532 nm laser excitation source to collect spectra in the range of 200 to 2000 cm^−1^. The microstructural and elemental characterization of the pre- and post-oxidation samples was conducted via scanning electron microscopy with energy-dispersive spectroscopy (SEM-EDS, Quanta 650 FEG, Thermo Fisher Scientific, Brno, Czech Republic). The analysis was carried out in secondary electron mode under conditions of a 20 kV accelerating voltage and a 3.5 μA beam current. For the nanoscale investigation of the oxide layer formed during oxidation, transmission electron microscopy (TEM, Talos F200x, Thermo Fisher Scientific, Waltham, MA, USA) coupled with EDS was utilized. The TEM specimen was prepared using a focused ion beam (FIB, Helios 5UC, Thermo Fisher Scientific, Waltham, MA, USA) via an in situ lift-out technique. Crystal analysis was performed by applying a fast Fourier transform (FFT) to the HRTEM images in DigitalMicrograph (version 3.40.2804.0). Data plotting was performed using OriginPro (version 2025b).

### 2.3. Isothermal Oxidation Tests on SiTiOC Ceramics

The high-temperature oxidation resistance of SiTiOC was evaluated through isothermal oxidation tests conducted at 1500 °C in air, using a horizontal corundum tube furnace (Hefei Kejing, Hefei, China). After oxidation, the samples were removed, cooled to room temperature, and weighed using a high-accuracy electronic balance (accuracy: ±0.00001 g). The mass change rate (Δ*m_i_%*) was calculated by the following formula:

Δ*m_i_*% = (*m*_0_ − *m_i_*)/*m*_0_ × 100% (1)

where *m*_0_ is the initial mass before oxidation, and *m_i_* is the mass after oxidation for *i* hours (where *i* = 2, 4, 6, 8, 16, and 32). For each composition (0Ti, 0.05Ti, 0.1Ti, and 0.2Ti), five separate samples were oxidized under identical conditions to ensure statistical reliability. The mass change data reported are the mean values, and the error bars represent the standard deviation.

## 3. Results

### 3.1. Structure Evolution of SiTiOC Ceramics

**Figure 1:**
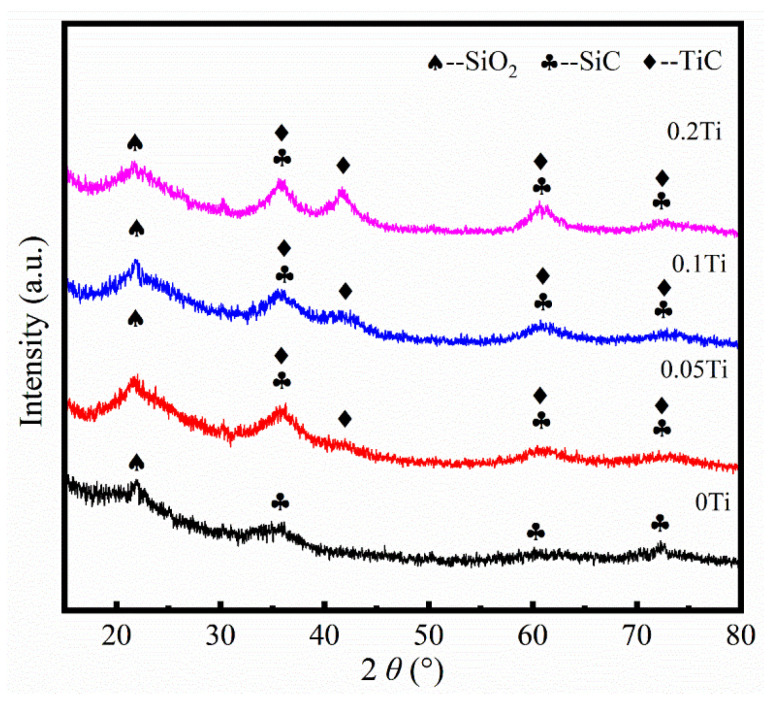
XRD patterns of SiTiOC ceramics.

Figure 1 presents the XRD patterns of SiTiOC ceramics with different Ti doping contents. A broad diffraction peak at 2*θ* = 21.9° was observed in all ceramics, which corresponds to the low-crystallinity SiO_2_. For the 0Ti ceramic, three diffraction peaks of 3C-SiC (2*θ* = 35.7°, 60.1°, and 72.1°; ICDD No. 00-029-1129) were also observed, indicating that the phase separation occurred above 1200 °C [21]. For Ti-doped ceramics, alongside the SiC phase, a TiC phase (2*θ* = 35.9°, 41.7°, 60.5°, and 72.4°; ICDD No. 00-032-1383) was also identified. The diffraction peak of TiC at 2*θ* = 41.7° was enhanced by increasing the Ti doping content, indicating that the TiC proportion increased.

**Figure 2:**
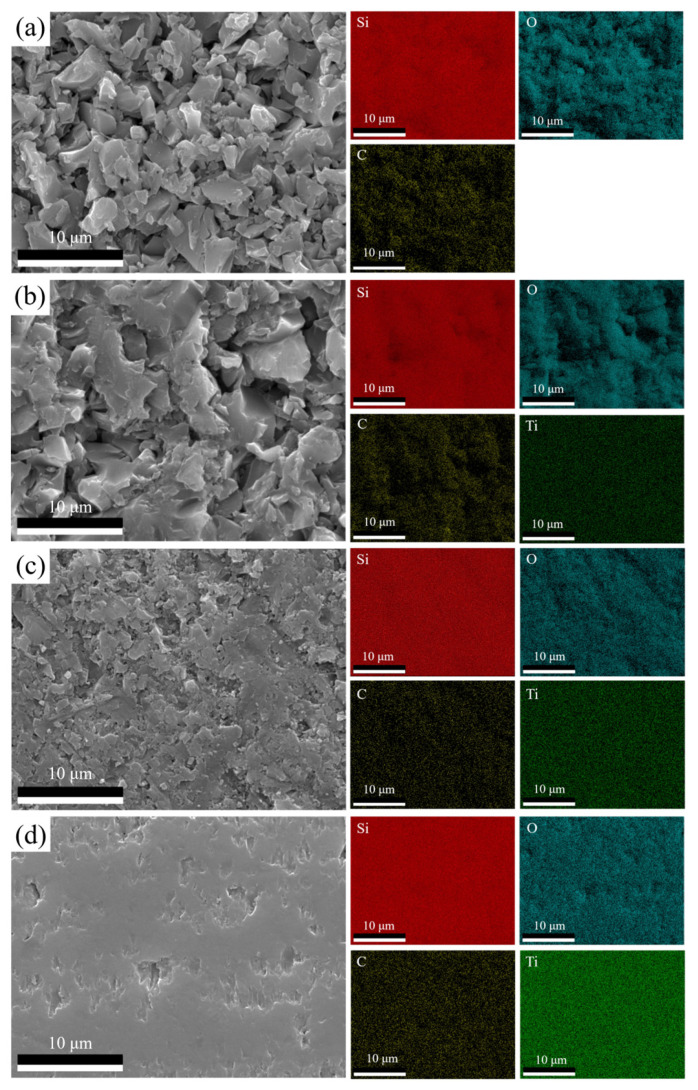
Surface morphologies and elemental distributions of SiTiOC ceramics: (**a**) 0Ti, (**b**) 0.05Ti, (**c**) 0.1Ti, and (**d**) 0.2Ti ceramics.

The surface morphologies and elemental distributions of SiTiOC ceramics with different Ti doping contents are shown in Figure 2. No obvious cracks were observed in these ceramic surfaces. Furthermore, the distributions of Si, O, C, and Ti elements were relatively uniform in the EDS results. However, the 0Ti ceramic exhibited a loose structure (Figure 2a), whereas the Ti-doped ceramics exhibited progressively denser structures with increased Ti doping contents (Figure 2a–d). The densities measured for 0Ti, 0.05Ti, 0.1Ti, and 0.2Ti ceramics were 1.76, 1.83, 2.06, and 2.45 g/cm^3^, respectively. The density changes were consistent with the ceramic morphologies. According to our previous work, Ti oxides such as TiO_2_ and Ti_2_O_3_ can be detected by XPS and increased with the concentration of Ti [20]. The viscosity of TiO_2_ is much lower than that of SiO_2_ at 1400 °C [22]. Therefore, TiO_2_ reduced the viscosity of the mixture, improved the wetting of particles (SiC and TiC) and accelerated the pore filling during the SPS process. As a result, the densification degree of the ceramics gradually increased with the Ti doping content.

### 3.2. High-Temperature Oxidation Behavior of SiTiOC Ceramics

**Figure 3:**
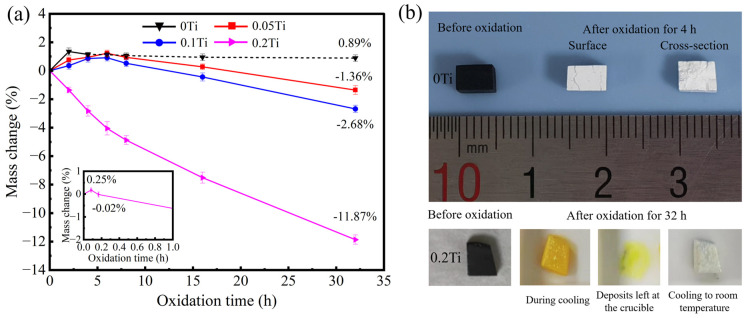
(**a**) Mass change curves of SiTiOC ceramics with varying Ti doping contents after oxidation in air at 1500 °C for 32 h; (**b**) optical image of 0Ti ceramic before and after oxidation for 4 h and the color change in 0.2Ti ceramics before and after oxidation for 32 h.

Figure 3a presents the mass change curves of SiTiOC ceramics subjected to oxidation in air at 1500 °C for 32 h. The complex mass fluctuation behavior originates from competing mechanisms. Specifically, the mass gain is induced by the passive oxidation of SiOC/SiC to SiO_2_ and the passive oxidation of TiC to TiO_2_. Conversely, the mass loss results from the active oxidation of these phases (which forms SiO and TiO) and the oxidation of free carbon. For the 0Ti ceramic, a mass gain of 1.35% was observed after 2 h, which subsequently decreased to 1.13% after 4 h. Thereafter, the mass continued to decline a little by 0.24% until 32 h. The corresponding optical images (Figure 3b) revealed significant morphological changes: the originally black 0Ti ceramic transformed to white within 4 h, with noticeable cracking. The observed color change in both the surface and cross-section indicated that the 0Ti ceramic underwent rapid oxidation to SiO_2_ within the first 4 h.

For the 0.05Ti and 0.1Ti ceramics, the mass increased initially by 1.22% and 0.91% at 6 h, and then it decreased gradually, with a mass loss of 1.36% and 2.68% at 32 h, respectively. Whilst for 0.2Ti, the mass increased only in the first 5 min by 0.25% from the enlarged local mass change curve. Subsequently, it decreased continuously, resulted in a mass loss of 11.87% after 32 h. Notably, the Ti-doped ceramics underwent a color change from golden yellow to white during the cooling process, especially for 0.2Ti (Figure 3b). When these samples were removed after oxidation, they appeared golden yellow at high temperatures (Figure 3b), and the deposits left at the crucible were also golden yellow. Combined with the literature reports by Voitovich [23], such golden-yellow volatile deposits correspond to titanium suboxide (TiO). Upon cooling to room temperature, both these samples and deposits at the crucible turned white, indicating that TiO was further oxidized to the stable, white TiO_2_.

**Figure 4:**
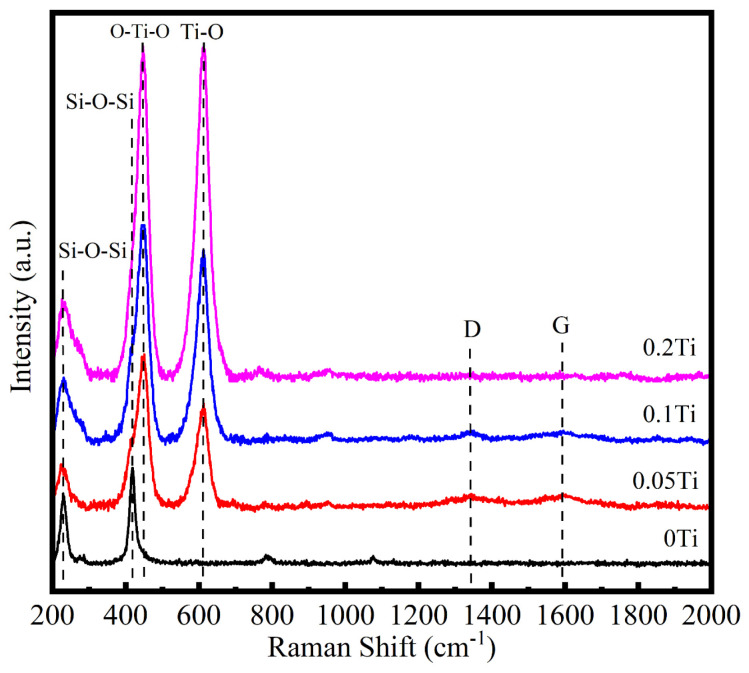
Raman spectra of SiTiOC ceramics after oxidation at 1500 °C for 2 h.

Raman spectra were produced to investigate the structural evolution of SiTiOC ceramics after oxidation at 1500 °C for 2 h, as shown in Figure 4. For the 0Ti ceramic, cristobalite SiO_2_ was formed after oxidation, with peaks at 228 cm^−1^ and 417 cm^−1^ corresponding to the bending and symmetrical stretching vibration modes of Si-O-Si, respectively [24]. In the Ti-doped ceramics, rutile TiO_2_ was also observed in addition to cristobalite SiO_2_, exhibiting two peaks at 447 cm^−1^ and 612 cm^−1^, which are associated with the bending and symmetrical stretching vibration modes of Ti-O-Ti, respectively [25]. No D or G bands related to free carbon were detected in the 0Ti ceramic, indicating that the free carbon was completely oxidized. In contrast, weak D (1345 cm^−1^) and G (1594 cm^−1^) bands were observed in the 0.05Ti and 0.1Ti ceramics, suggesting that small amounts of free carbon remained after 2 h of oxidation. However, in the 0.2Ti ceramic, no D or G bands were observed, confirming that the free carbon had also been oxidized.

**Figure 5:**
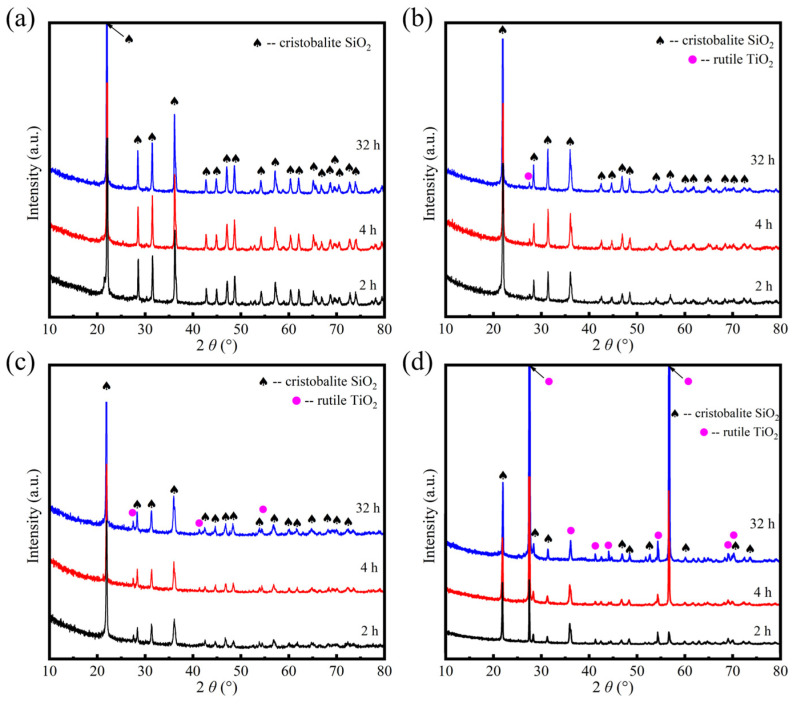
XRD patterns of SiTiOC ceramics after oxidation in air at 1500 °C. (**a**) 0Ti, (**b**) 0.05Ti, (**c**) 0.1Ti, and (**d**) 0.2Ti ceramics.

**Figure 6:**
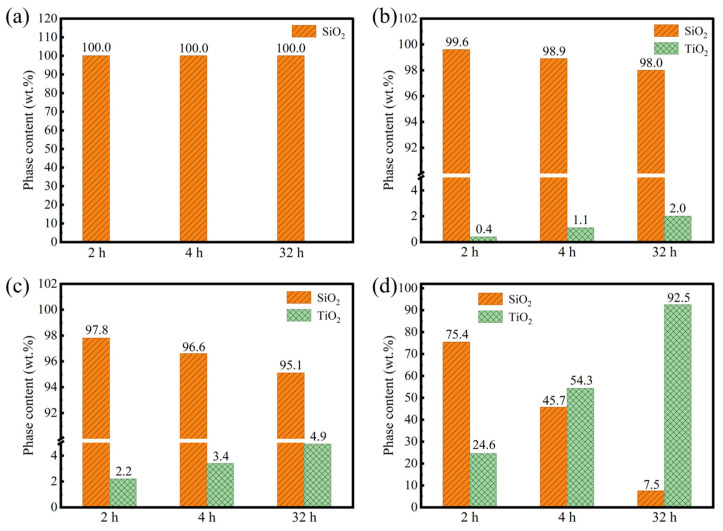
Phase content of SiTiOC ceramics after oxidation in air at 1500 °C. (**a**) 0Ti, (**b**) 0.05Ti, (**c**) 0.1Ti, and (**d**) 0.2Ti ceramics.

An XRD analysis was performed on the oxidized SiTiOC ceramics to investigate the evolution of the phase composition during oxidation, as shown in Figure 5. The corresponding quantitative phase content, analyzed to track the oxidation progress, is presented in Figure 6. For the 0Ti ceramic, diffraction peaks associated with the cristobalite SiO_2_ (ICDD No. 00-039-1425) phase were observed (Figure 5a). No crystalline phases other than SiO_2_ were detected throughout the entire oxidation process. The intensity of the main SiO_2_ peak at 22.0° enhanced slightly from 2 h to 4 h, indicating the growth of the silica layer. For the oxidation for 32 h, the diffraction peaks had barely changed. This phenomenon confirmed that 0Ti has been oxidized completely after 4 h, consistent with preceding morphological observations (Figure 3b).

For 0.05Ti ceramics, the cristobalite SiO_2_ peaks were still dominant and intensified with prolonged oxidation. At the same time, a weak diffraction peak of the rutile TiO_2_ (ICDD No. 00-021-1276) phase was observed at 27.5° after oxidation for 2 h and enhanced slightly after oxidation for 4 h. The content of the TiO_2_ phase gradually increased from 0.4% at 2 h to 2.0% after 32 h (Figure 6b). A similar trend was observed for the 0.1Ti ceramic, where the final TiO_2_ content reached 4.9% after 32 h (Figure 6c), indicating a slow but continuous formation of titania within the silica-dominated layer. A marked transition occurred at higher Ti doping contents. For the 0.2Ti ceramic, the rutile TiO_2_ peaks were pronounced after only 2 h of oxidation, with a content of 24.6% (Figure 6d). After 4 h, the intensity of the main TiO_2_ peak exceeded that of the cristobalite SiO_2_ (Figure 5d), corresponding to a dramatic increase in the TiO_2_ content to 54.3%. Notably, the TiO_2_ content reached 92.5% after 32 h, demonstrating that the oxide layer was overwhelmingly composed of TiO_2_. In summary, the oxidation pathway shifts fundamentally with Ti doping. The undoped ceramic is fully consumed, transforming into a SiO_2_ within 4 h. Low Ti doping (≤0.1Ti) slows this process, yielding an incomplete, mixed oxide. Whereas, for 0.2Ti the oxidation remains rapid and produces a dominant TiO_2_ layer. This switch from silica-forming to titania-forming during oxidation dictates the overall oxidation resistance.

**Figure 7:**
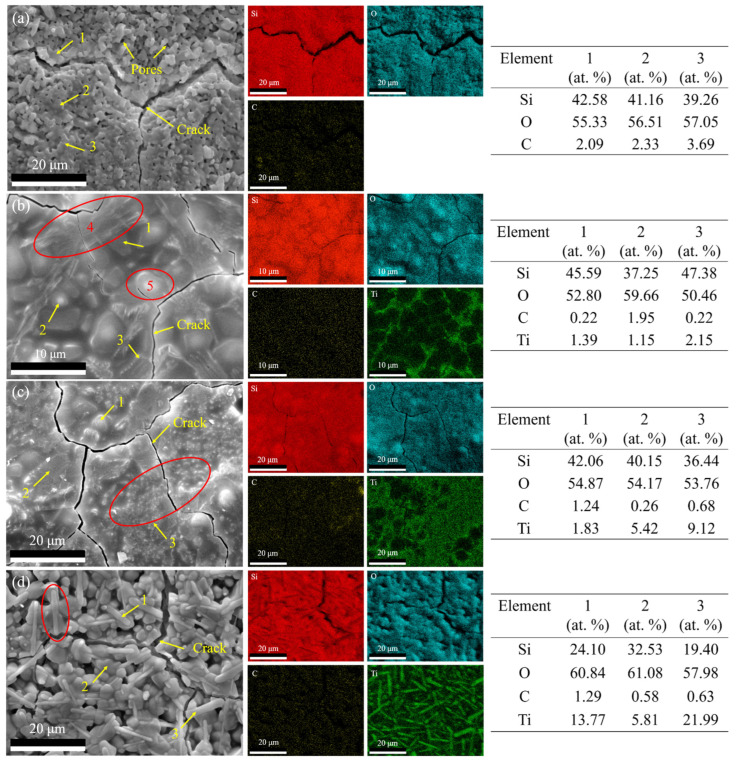
Surface morphologies and elemental distribution of SiTiOC ceramics after oxidation for 2 h. (**a**) 0Ti, (**b**) 0.05Ti, (**c**) 0.1Ti, and (**d**) 0.2Ti ceramics.

The surface morphologies and elemental distribution of SiTiOC ceramics after oxidation at 1500 °C for 2 h (Figure 7) were analyzed by SEM-EDS. The 0Ti ceramic exhibited large amounts of pores (Figure 7a), attributed to the substantial release of gaseous CO_x_ and SiO during oxidation. Additionally, surface cracking was primarily ascribed to the volume contraction induced by the phase transformation from amorphous SiO_2_ to cristobalite SiO_2_, and the passive oxidation of SiC/SiOC generated cristobalite SiO_2_. Both phenomena facilitated rapid oxygen diffusion, thereby exacerbating the internal oxidation of the ceramics. The EDS results showed that the surface mainly contained Si and O elements. And the C detected for 0Ti was likely due to the measurement artifacts, as no free carbon was observed in the Raman spectra (Figure 4).

For the 0.05Ti and 0.1Ti ceramics, the surfaces exhibited flat and compact morphologies with only small cracks and negligible porosity (Figure 7b,c). Pebble-like particles (the red circled area 4 in Figure 7b) and dendritic particles (the red circled area 5 in Figure 7b) were observed on the surface of the 0.05Ti ceramic. EDS results revealed that the pebble-like particles were rich in Si, whilst the dendritic particles were rich in Ti (Figure 6b). TiO_2_ was observed to segregate at the SiO_2_ grain boundaries (Figure 7b) in a network-like distribution. This occurs because the high-energy grain boundaries act as rapid diffusion pathways for Ti cations, as reported by Lapington et al. [26] and Zschiesche et al. [27]. For the 0.1Ti ceramic, the size of Si-rich pebble-like particles decreased (Figure 7c), and numerous fine, raised Ti-rich particles appeared (the red circled area in Figure 7c). In contrast, the surface of 0.2Ti exhibited not only pronounced cracks but also abundant porosity (Figure 7d). Meanwhile, the Si-rich pebble-like particles reduced in size (Figure 7d), and numerous Ti-rich elongated rod-like particles were observed, as indicated in the red-circled regions of Figure 7d.

**Figure 8:**
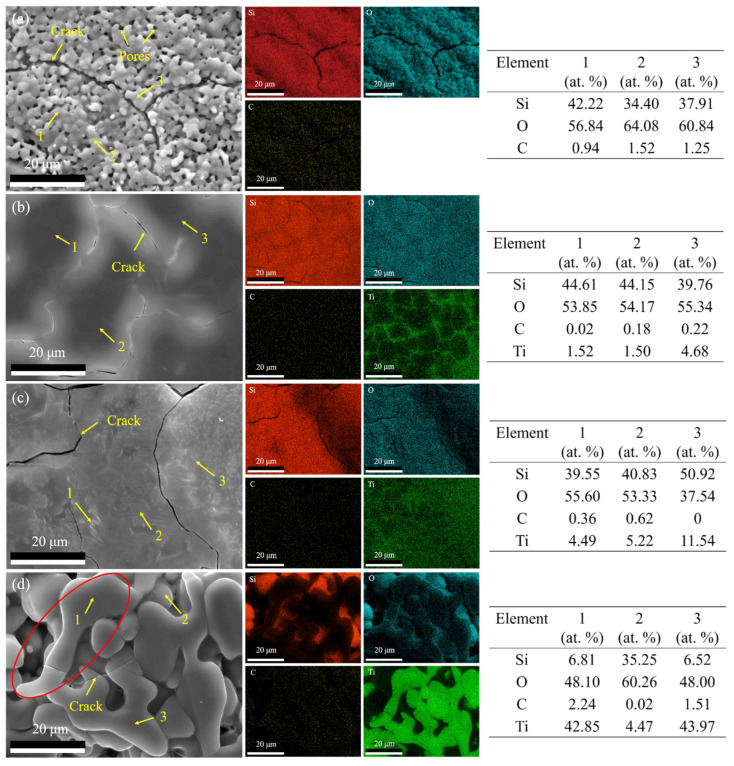
Surface morphologies and elemental distribution of SiTiOC ceramics after oxidation for 32 h. (**a**) 0Ti, (**b**) 0.05Ti, (**c**) 0.1Ti, and (**d**) 0.2Ti ceramics.

The surface morphologies and elemental distribution of the SiTiOC ceramics following the oxidation at 1500 °C for 32 h are presented in Figure 8. The 0Ti ceramic maintained significant surface cracking along with markedly increased porosity (Figure 8a). For the 0.05Ti ceramic, the surface became flatter with narrower cracks and maintained the reticulated Ti segregation pattern. For 0.1Ti, the surface became smoother with little crack changes. The fine Ti-rich particles were distributed throughout the surface, while the Si-rich pebble-like particles disappeared, whereas the surface of the 0.2Ti ceramic was irregular with pits and small cracks. Furthermore, the surface exhibited large-sized irregular Ti-rich particles (the red circled area in Figure 8d), which resulted from the growth and fusion of long rod-like particles (Figure 7d) during oxidation. The elemental distribution of Si and Ti exhibited an inverse correlation with clear boundaries. Point scanning results indicated that the Ti content on the surface increased with both the Ti doping content and the duration of the oxidation.

**Figure 9:**
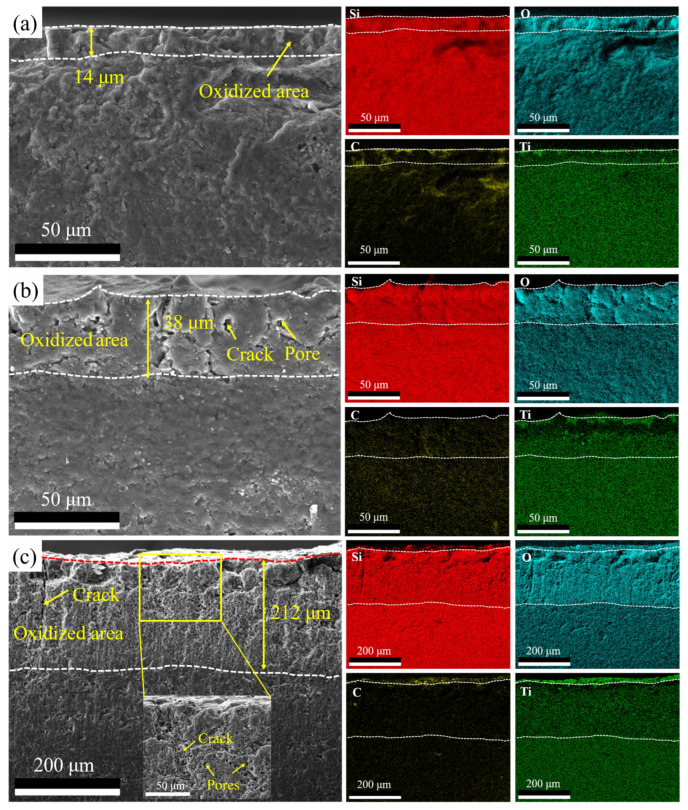
Cross sectional morphologies and elemental distribution of SiTiOC ceramics after oxidation for 32 h. (**a**) 0.05Ti, (**b**) 0.1Ti, and (**c**) 0.2Ti ceramics.

The cross-sectional morphologies and elemental distribution of the SiTiOC ceramics after oxidation for 32 h are shown in Figure 9. No obvious defects were observed in the cross-sectional morphology of the 0.05Ti ceramic (Figure 9a), with an oxide layer thickness of 14 μm. For the 0.1Ti ceramic, the oxide layer exhibited some pores and cracks, with a thickness of 38 μm (Figure 9b). For the 0.2Ti ceramic, not only cracks but large amounts of pores were observed within the oxide layer (enlarged SEM image of the rectangular area in Figure 9c), and the oxide layer thickness increased to 212 μm. Note that in the 0.1Ti and 0.2Ti ceramics, Ti-poor layers were observed within the oxide layer, as well as Ti element aggregation on the surface (Figure 7 and Figure 8). This phenomenon can be ascribed to the two following reasons: (1) the outward diffusion rate of Ti^4+^ is faster than other cations like Si^4+^ [28,29,30,31,32], and (2) TiO_2_ is less susceptible to evaporation or decomposition at 1500 °C [22].

**Figure 10:**
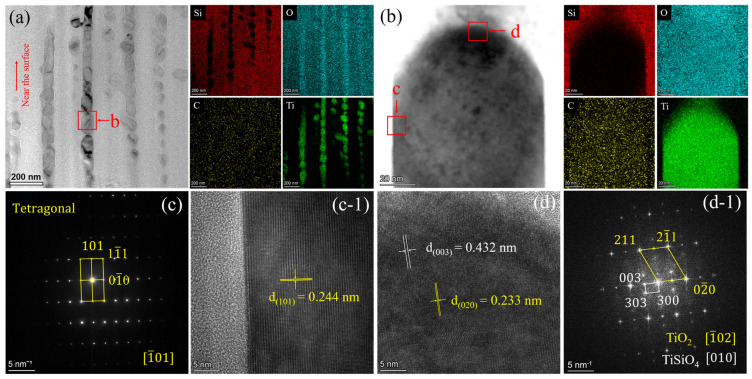
TEM images of cross-section of oxide layer of 0.05Ti ceramic bulk after oxidation for 32 h: (**a**) BF image and EDS mapping; (**b**) enlarged BF image and EDS mapping of region b in (**a**); (**c**,**c-1**) SAED pattern and HRTEM image of region c in (**b**); and (**d**,**d-1**) HRTEM and FFT image of region d in (**b**).

A TEM analysis was conducted on the cross-section of the oxide layer of the 0.05Ti ceramic after oxidation for 32 h (Figure 10). The bright-field (BF) image (Figure 10a) revealed a significant number of grains oriented longitudinally. The EDS results (Figure 10a,b) indicated that these grains were rich in Ti. The selected area electron diffraction (SAED) pattern (Figure 10c) and high-resolution TEM (HRTEM) image (Figure 10(c-1)) identified for the grain was rutile TiO_2_ with a tetragonal structure, with (101) an interplanar spacing of 0.244 nm recorded along the [$\bar{1}$01] zone axis. Furthermore, a coexistence zone of Si and Ti was observed at the upper edge of the grain (region d of Figure 10b), where TiSiO_4_ was also identified through the HRTEM and fast Fourier transform (FFT) images (Figure 10(d,d-1)). In region d, TiO_2_ was recorded along the [$\bar{1}$02] zone axis, with 0.233 nm interplanar spacing for the (020) plane; the TiSiO_4_ recorded along the [010] zone axis had 0.432 nm interplanar spacing for the (003) plane. The formation of TiSiO_4_ indicated that interfacial reactions between outward-diffusing TiO_2_ and SiO_2_ were driven by the high reactivity along the diffusion pathways of TiO_2_.

## 4. Discussion

**Figure 11:**
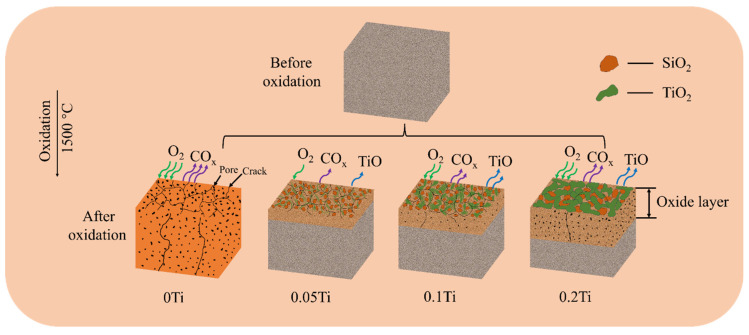
Schematic diagram of oxidation behavior of SiTiOC ceramics.

The experimental results clearly demonstrated that the incorporation of Ti could significantly enhance the oxidation resistance of SiOC ceramics. In particular, the 0.05Ti ceramic exhibited the optimal oxidation resistance. Beyond this critical doping threshold, the oxidation resistance of these ceramics gradually deteriorates with increased Ti doping contents, as illustrated in the proposed oxidation mechanism schematic (Figure 11). This phenomenon is closely associated with the structural integrity and chemical stability of the surface oxide scale.

### 4.1. Oxidation Mechanism of Undoped Ceramic (0Ti)

For the 0Ti ceramic, the oxidation process at 1500 °C in air can be described by the following reactions:

SiO_x_C_4−x_ (s) + O_2_ (g) → SiO_2_ (s) + CO_x_ (g) (2)

SiC (s) + O_2_ (g) → SiO_2_ (s) + CO_x_ (g) (3)

C (s) + O_2_ (g) →CO_x_ (g) (4)

SiC (s) + O_2_ (g) → SiO (g) + CO_x_ (g) (5)

SiO_x_C_4−x_ (s) + O_2_ (g) → SiO (g) + CO (g) (6)

SiO_2_ (s) → SiO (g) + O_2_ (g) (7)

The 0Ti ceramic with a loose structure (Figure 2a) initially exhibited rapid passive oxidation (Equations (2) and (3)), resulting in SiO_2_ formation and CO_x_ volatilization that caused mass gains (≤2 h), whilst the concurrent oxidation of free carbon (Equation (4)) and active oxidation (Equations (5) and (6)) produced gaseous species like SiO and CO_x_, which led to mass loss. Although SiO_2_ provided partial surface protection in the early stage, the excessive gas evolution compromised the integrity of the oxide layer, which permitted continuous oxygen ingress into the ceramic matrix. This permeable oxide layer enabled the matrix to be completely oxidized within 4 h (Figure 3b) as well as a small mass loss due to the decomposition of minor SiO_2_ (Equation (7)). Ultimately, the 0Ti ceramic demonstrated poor oxidation resistance.

### 4.2. Ti-Doped Systems: Performance Optimization and Degradation Mechanisms

For Ti-doped ceramics, in addition to the above reactions (Equations (2)–(7)), the following reactions regarding Ti also occur during the oxidation process in air at 1500 °C:

TiC (s) + O_2_ (g) → TiO_2_ (s) + CO_x_ (g) (8)

TiOC (s) + O_2_ (g) → TiO_2_ (s) + CO_x_ (g) (9)

Ti_2_O_3_ (s) + O_2_ (g) → TiO_2_ (s) (10)

TiC (s) + O_2_ (g) → TiO (g) + CO (g) (11)

TiOC (s) + O_2_ (g) → TiO (g) + CO (g) (12)

The 0.05Ti ceramic with a relatively loose structure (Figure 2b) initially exhibited rapid oxygen diffusion. The ceramic was oxidized (Equations (2)–(4) and (8)), forming an oxide layer (Figure 7b and Figure 8b) dominated by SiO_2_ with trace amounts of TiO_2_ (Figure 5b). SEM images (Figure 7b and Figure 8b) showed that TiO_2_ segregated at the grain boundaries of SiO_2_. Generally, these boundaries serve as fast pathways for oxygen diffusion but were effectively stabilized by the Ti^4+^ enrichment, which reduced the interfacial Gibbs free energy and hindered the oxygen ingress [33]. The TiSiO_4_ formed at the interfaces between SiO_2_ and TiO_2_ (Figure 10d) may have further stabilized the oxide layer, which is similar to ZrSiO_4_ and HfSiO_4_ in Zr/Hf-doped ceramics [34]. Furthermore, the network-like distribution of TiO_2_ at the grain boundaries of SiO_2_ played a beneficial role in crack pinning and inhibited the further expansion of cracks [34]. Consequently, the composite oxide layer of the 0.05Ti ceramic demonstrated an enhanced stability with a small oxygen diffusion coefficient and strong self-healing capability. The 0.05Ti ceramic showed a mass gain within 6 h under the dominance of the passive oxidation of SiC, SiOC, and TiC. With the extension of the oxidation time, the slow active oxidation (Equations (5), (6) and (9)) of the ceramic matrix became predominant (≥6 h), attributed to the reduced oxygen partial pressure caused by the obstruction of the oxide layer. Notably, the active oxidation of TiC occurred prior to that of SiC [22], and the mass loss (≥6 h) was mainly caused by the volatilization of the gaseous gold–yellow TiO.

As the Ti doping content increased, the 0.1Ti ceramic with a relatively compact structure (Figure 2c) initially exhibited reduced oxygen diffusion pathways. The ceramic underwent oxidation and formed a relatively compact oxide layer. However, the outward diffusion of Ti was enhanced as the Ti doping content increased, which caused damage to the oxide layer. This damage accelerated the outward diffusion of C and the inward diffusion of O^2−^ [35,36], thereby aggravating the oxidation of the internal ceramic matrix (passive oxidation and active oxidation). The quantities of TiO_2_ and TiO generated increased with Ti doping levels. Nevertheless, the diffusion rate of the oxygen increased with the rising TiO_2_ content of the oxide layer, as the diffusion rate of oxygen in TiO_2_ is much higher than that in SiO_2_ [22]. Concurrently, the volatilization of gaseous TiO intensified, further compromising the integrity of the oxide layer. As the oxidation proceeded, the oxide layer thickened and formed cracks (Figure 9b) due to the fact that the thicker oxide layer stores greater thermal mismatch strain [37]. Consequently, the oxidation resistance of the 0.1Ti ceramic deteriorated. The 0.1Ti ceramic showed a mass gain within the first 6 h, followed by an increase in the mass loss due to the increased volatilization of TiO (≥6 h).

By further increasing the Ti doping content, although the 0.2Ti ceramic had the highest density with a compact structure (Figure 2d), the active oxidation of TiC (Equation (9)) in the internal ceramic matrix commenced rapidly, which led to the degradation of the oxide layer from the release of TiO and an observable mass loss after merely 5 min of oxidation. The significantly enhanced outward diffusion of Ti and the ongoing volatilization of the generated TiO resulted in a more porous and looser oxide layer with internal cracks (Figure 9c). Consequently, the structure of the oxide layer was constantly damaged with reduced stability. Ultimately, the oxidation resistance of the 0.2Ti ceramic further deteriorated.

## 5. Conclusions and Outlook

The high-temperature oxidation behavior of SiTiOC ceramics with varying Ti doping contents in air at 1500 °C for 32 h has been investigated. The effect of Ti-doping on the oxidation behavior of SiTiOC ceramics was systematically analyzed. Based on the above results and discussions, the main conclusions are summarized as follows:

(1) The SiOC ceramic with a loose structure underwent severe oxidation, with released substantial gaseous byproducts at 1500 °C, and was completely oxidized within 4 h. In contrast, the incorporation of Ti into SiOC can improve the oxidation resistance for the formed effective oxide layer.

(2) The 0.05Ti ceramic bulk demonstrated the best oxidation resistance, with a mass loss of 1.36% after oxidation for 32 h. This was mainly attributed to the development of a complete and compact composite oxide layer, which was composed primarily of SiO_2_, with minor amounts of TiO_2_ segregated the grain boundaries. This oxide layer exhibited a low oxygen diffusion rate and strong self-healing capability.

(3) As the Ti doping content increased, the significant outward diffusion of Ti accelerated the active oxidation of the internal ceramic matrix and damaged the integrity of the oxide layer. Furthermore, the oxygen diffusion rate of the oxide layer increased with the increase in TiO_2_. Consequently, the oxidation resistance of the ceramics diminished with increased Ti-doped contents.

While this work identifies an optimal Ti/Si ratio (~0.05) for oxidation resistance at 1500 °C, translating this finding into practical applications requires further development. We should evaluate performances under realistic service conditions, such as thermal cycling, water vapor/salt-containing atmospheres, and long-term aging (>500 h), and characterize essential application-specific properties, like thermal conductivity, the thermal expansion coefficient, and high-temperature mechanical strength. Future work should also explore co-doping strategies (e.g., Ti with Al/Zr) and processing into applicable forms (e.g., coatings). Ultimately, proof-of-concept validation in simulated environments is needed to demonstrate technological viability for aerospace applications.

## Data Availability

The original contributions presented in this study are included in the article. Further inquiries can be directed to the corresponding authors.

## References

[1] Stabler C., Ionescu E., Graczyk-Zajac M., Gonzalo-Juan I., Riedel R. (2018). Silicon oxycarbide glasses and glass-ceramics: “All-Rounder” materials for advanced structural and functional applications. J. Am. Ceram. Soc..

[2] Colombo P., Mera G., Riedel R., Sorarù G.D. (2010). Polymer-Derived Ceramics: 40 Years of Research and Innovation in Advanced Ceramics. J. Am. Ceram. Soc..

[3] Yu S., Tu R., Goto T. (2016). Preparation of SiOC nanocomposite films by laser chemical vapor deposition. J. Eur. Ceram. Soc..

[4] Yan J., Wu X., Liu Q., Li Y., Yu S. (2024). Microstructure and oxidation behavior of SiOC coatings on C/C composites co-deposited with HMDS and TEOS by using CVD process. Ceram. Int..

[5] Yan H.-J., Li Y.-Y., Yin R.-Z., Sun Q.-Q., Liu H.-J., Zeng C.-L., Wu L.-K., Cao F.-H. (2023). High temperature oxidation behavior of TiAl alloy with electrodeposited SiOC coating. Corros. Sci..

[6] Zhou S., Yao L., Zhao T., Mei H., Cheng L., Zhang L. (2022). Ti doped SiOC precursor to activate gyroid sensing structures. Carbon.

[7] Yang N., Lu K. (2019). Thermophysical property and electrical conductivity of titanium isopropoxide—Polysiloxane derived ceramics. J. Eur. Ceram. Soc..

[8] Saha A., Raj R. (2007). Crystallization Maps for SiCO Amorphous Ceramics. J. Am. Ceram. Soc..

[9] Zhou Y., Guo L., Ma Q., Dong S., Mao W. (2022). Effect of Hafnium content on structural evolution of SiHfOC ceramics at high temperatures. Ceram. Int..

[10] Xu T., Ma Q., Chen Z. (2011). High-temperature behavior of C_f_/SiOC composites in inert atmosphere. Mater. Sci. Eng. A.

[11] Xu T., Ma Q., Chen Z. (2011). The effect of aluminum additive on structure evolution of silicon oxycarbide derived from polysiloxane. Mater. Lett..

[12] Hu Z., Guo L., Ma Q. (2020). Effects of yttrium on structure evolution of silicon oxycarbide derived from polysiloxane. Ceram. Int..

[13] Zhang X., Wang B., Wu N., Han C., Wang Y. (2021). Multi-phase SiZrOC nanofibers with outstanding flexibility and stability for thermal insulation up to 1400 °C. Chem. Eng. J..

[14] Ionescu E., Linck C., Fasel C., Müller M., Kleebe H., Riedel R. (2010). Polymer-Derived SiOC/ZrO_2_ Ceramic Nanocomposites with Excellent High-Temperature Stability. J. Am. Ceram. Soc..

[15] Zhang X. (2020). Flexible and thermal-stable SiZrOC nanofiber membranes with low thermal conductivity at high-temperature. J. Eur. Ceram. Soc..

[16] Sun J., Wen Q., Li T., Wiehl L., Fasel C., Feng Y., De Carolis D., Yu Z., Fu Q., Riedel R. (2020). Phase evolution of SiOC-based ceramic nanocomposites derived from a polymethylsiloxane modified by Hf- and Ti-alkoxides. J. Am. Ceram. Soc..

[17] Ionescu E., Papendorf B., Kleebe H., Riedel R. (2010). Polymer-Derived Silicon Oxycarbide/Hafnia Ceramic Nanocomposites. Part II: Stability Toward Decomposition and Microstructure Evolution at T≫1000 °C. J. Am. Ceram. Soc..

[18] Sun J., Li T., Reitz A., Fu Q., Riedel R., Yu Z. (2020). High-temperature stability and oxidation behavior of SiOC/HfO_2_ ceramic nanocomposite in air. Corros. Sci..

[19] Sun X., Yang G., Tian Z., Zhu W., Su D. (2022). In-situ formation of titanium carbide in carbon-rich silicon oxycarbide ceramic for enhanced thermal stability. J. Eur. Ceram. Soc..

[20] Wu X., Li Y., Chen X., Yu S. (2025). Influence of Ti addition on the structure, thermal stability of SiTiOC ceramics and pyrolysis behavior of SiTiOC xerogel. Ceram. Int..

[21] Stabler C., Celarie F., Rouxel T., Limbach R., Wondraczek L., Riedel R., Ionescu E. (2019). Effect of composition and high-temperature annealing on the local deformation behavior of silicon oxycarbides. J. Eur. Ceram. Soc..

[22] Pan X., Niu Y., Xu X., Zhong X., Shi M., Zheng X., Ding C. (2020). Long time ablation behaviors of designed ZrC-SiC-TiC ternary coatings for environments above 2000 °C. Corros. Sci..

[23] Voitovich V.B. (1997). Mechanism of the High Temperature Oxidation of Titanium Carbide. High Temp. Mater. Process..

[24] Sigaev V.N., Smelyanskaya E.N., Plotnichenko V.G., Koltashev V.V., Volkov A.A., Pernice P. (1999). Low-frequency band at 50 cm^−1^ in the Raman spectrum of cristobalite: Identification of similar structural motifs in glasses and crystals of similar composition. J. Non-Cryst. Solids.

[25] Rani C., Pathak D.K., Tanwar M., Kandpal S., Ghosh T., Maximov M.Y., Kumar R. (2022). Anharmonicity induced faster decay of hot phonons in rutile TiO_2_ nanorods: A Raman spectromicroscopy study. Mater. Adv..

[26] Lapington M.T., Crudden D.J., Reed R.C., Moody M.P., Bagot P.A.J. (2021). Characterization of oxidation mechanisms in a family of polycrystalline chromia-forming nickel-base superalloys. Acta Mater..

[27] Zschiesche H., Charaï A., Mangelinck D., Alfonso C. (2019). Ti segregation at CoSi_2_ grain boundaries. Microelectron. Eng..

[28] Lee D.B., Park S.W. (2007). Oxidation of Ti_3_SiC_2_ between 900 and 1200 °C in Air. Oxid. Met..

[29] Teng J., Gong X., Yang B., Yu S., Liu J., Li Y. (2021). Influence of Ti addition on oxidation behavior of Ni-Cr-W-based superalloys. Corros. Sci..

[30] Ye X., Yang B., Nie Y., Yu S., Li Y. (2021). Influence of Nb addition on the oxidation behavior of novel Ni-base superalloy. Corros. Sci..

[31] Lu L., Wen T., Li W., Wen Q., Yu Z., Tao S., Yang J., Wang Y., Luan X., Xiong X. (2024). Single-source-precursor synthesis of dense monolithic SiC/(Ti_0.25_Zr_0.25_Hf_0.25_Ta_0.25_)C ceramic nanocomposite with excellent high-temperature oxidation resistance. J. Eur. Ceram. Soc..

[32] Peng Z., Sun W., Xiong X., Zhang H., Guo F., Li J. (2021). Novel refractory high-entropy ceramics: Transition metal carbonitrides with superior ablation resistance. Corros. Sci..

[33] Gao S., He B., Zhou L., Hou J. (2020). Effects of Ta on the high temperature oxidation behavior of IN617 alloy in air. Corros. Sci..

[34] Liu B., Sun J., Guo L., Shi H., Feng G., Feldmann L., Yin X., Riedel R., Fu Q., Li H. (2025). Materials design of silicon based ceramic coatings for high temperature oxidation protection. Mater. Sci. Eng. R Rep..

[35] Chen S., Chen Z., Wang J., Zeng Y., Song W., Xiong X., Li X., Li T., Wang Y. (2024). Insight into the effect of Ti substitutions on the static oxidation behavior of (Hf,Ti)C at 2500 °C. Adv. Powder Mater..

[36] Pedrazzini S., Rowlands B.S., Turk A., Parr I.M.D., Hardy M.C., Bagot P.A.J., Moody M.P., Galindo-Nava E., Stone H.J. (2019). Partitioning of Ti and Kinetic Growth Predictions on the Thermally Grown Chromia Scale of a Polycrystalline Nickel-Based Superalloy. Metall. Mater. Trans. A.

[37] Niu M., Zhao Z., Su L., Gao H., Cai Z., Wang H. (2020). Oxidation behavior of dense SiOC monolithics: The oxide scale development. Corros. Sci..

